# Live Fish in the Throat Causing Upper Airway Obstruction and Esophageal Perforation

**DOI:** 10.7759/cureus.69316

**Published:** 2024-09-13

**Authors:** Jing Hui Fu, Sheih Nee Loke, Shong Sheng Tan, Nurhani Yasmin Abdul Rahman, Sze Li Siow

**Affiliations:** 1 Faculty of Medicine, University of Malaya, Kuala Lumpur, MYS; 2 Department of General Surgery, Sarawak General Hospital, Kuching, MYS; 3 Department of Surgery, Universiti Sains Malaysia, Kubang Kerian, MYS; 4 Department of Otolaryngology, Columbia Asia Hospital, Bintulu, MYS; 5 Department of Otolaryngology, Sarawak General Hospital, Kuching, MYS; 6 Department of Surgery, Taylor's University School of Medicine, Kuala Lumpur, MYS; 7 Faculty of Medicine, Universiti Malaysia Sarawak, Kota Samarahan, MYS

**Keywords:** acute airway obstruction, cervical esophageal injury, live fish in the throat, pneumomediastinum, subcutaneous emphysema, traumatic esophageal perforation, treatment of foreign body ingestion

## Abstract

Having a large live fish stuck in the throat is rare and prompts the urgent need to secure a definitive airway. Such sizeable foreign body (FB) in the aerodigestive tract also poses a constant threat of hollow viscus perforation, and the removal process can be complex.

This report describes a fishing mishap causing the impaction of a large live fish in the laryngopharynx and esophagus, leading to respiratory distress and upper esophageal perforation. The paper highlights the mechanism of injury, emergent airway management, anatomical consideration of the location of the FB, and technical challenges in FB removal. After endotracheal intubation, the depth and location of the live fish were confirmed with a plain radiograph. The removal of the live fish was eventually successful after dislodging its fins from the laryngopharynx and rotating its head out from the upper esophagus endoscopically. The upper esophageal perforation healed with non-operative management, and the patient was discharged well.

## Introduction

Accidental ingestion of foreign bodies (FBs) is a common complaint encountered in emergency and otolaryngologic practice [1,2]. Among adults, sharp objects like fish bones are the most commonly implicated FBs [1,2]. Patients typically present within a day of the incident, and the most frequent clinical presentations include odynophagia, dysphagia, and foreign sensation at the throat [1,2]. The majority of cases involve smaller FBs, which often pass through the gastrointestinal tract spontaneously [2]. However, some FBs can get impacted in the throat, which can usually be retrieved with the aid of a laryngoscopy [1,2]. They are often identified at the oropharyngeal area like the root of the tongue and at the epiglottic vallecula [1].

The impact of such incidents varies depending on the characteristics, shape, size, and nature of the FBs [1,2]. While such incidents can involve a wide variety of FBs, ingestion of a whole live fish is rare [3-10]. Only a handful of cases had been reported worldwide [3-10]. As sizeable FBs, live fishes often get impacted in the pharynx causing airway obstruction [3-10]. Spontaneous expulsion rarely occurred [6]. While Tang et al. and Saha et al. had described the successful removal of the impacted live fish after a timely, life-saving tracheostomy, most pass away due to failure of prompt FB removal and subsequent asphyxiation [3,5,8,9]. 

When lodged in the esophagus, the passage of FB can lacerate and breach the esophageal mucosa, causing esophageal perforation [2,11-16]. This is a life-threatening condition in which the extraluminal leakage of esophageal contents results in retropharyngeal abscess, sepsis, mediastinitis, and death [11-16]. Most patients present with nonspecific presentations including fever, odynophagia, and neck crepitus [11-16]. Due to its insidious and varying clinical presentations, its diagnosis is often delayed, posing a complex challenge for the planning of surgical intervention [11-16]. Its mortality rate ranges from 13.3% to 60%, depending on the time of onset to diagnosis, its etiology, and the part of the esophagus involved [11-15]. The perforation site can be classified into three categories: cervical, thoracic, and abdominal esophageal perforation [11-15]. Among these, cervical perforation is associated with the lowest mortality [11-15]. Treatment strategies include fluid resuscitation, antibiotic therapy for sepsis control, as well as consideration for surgical repair [11-16]. 

To date, no cases have described the accidental ingestion of a live fish causing the dual pathology of airway and esophageal injury. We report a case of the accidental ingestion of a large live fish after a fishing mishap. This case describes the importance of understanding the mechanism of injury and anticipating the need for emergent airway management. Such a case also highlights the need for prompt interdisciplinary coordination to tackle the technical challenges in the removal of the live fish and the consideration for the therapeutic options in managing cervical esophageal perforation. 

The emergent airway management part of this case was discussed as an abstract and an oral presentation at the European Airway Congress in Turkey in 2022.

## Case presentation

A middle-aged fit fisherman experienced neck discomfort and dyspnea after a live fish was lodged in his throat. While fishing with a rod, he reeled out a river fish, which rapidly broke out of the water and propelled directly into his mouth. The unexpected event led to subsequent struggles as the fish propagated further down his throat.

The patient arrived at the district non-specialist hospital within 10 minutes of the incident. Upon presentation, a loud, conspicuous flapping sound can be heard emanating from his neck. During brief pauses between the flapping sounds, the patient can speak complete sentences with clear articulation. However, visual inspection of the oropharynx failed to identify any FB.

The patient initially appeared restless due to throat pain but maintained a good oxygen saturation level under room air. However, as clinical assessment progressed, the previously audible flapping sound diminished. The patient subsequently developed harsh stridor and excessive bloody salivation. A direct laryngoscope was immediately introduced to secure a definitive airway.

Using a Macintosh blade size 4 (Mac 4) laryngoscope, a large fish tail was observed wriggling and intermittently obstructing the laryngeal inlet. It occupied nearly the entire laryngopharynx. The body and head of the fish were deeply embedded in the esophagus and remained beyond visual confirmation. A pair of curved forceps was used to maneuver the fish's tail away from the larynx to allow a clear view of the glottis. Subsequently, with the aid of stylet keeping the tube stiffened and unaffected by the moving tail, endotracheal intubation was performed smoothly.

**Figure 1:**
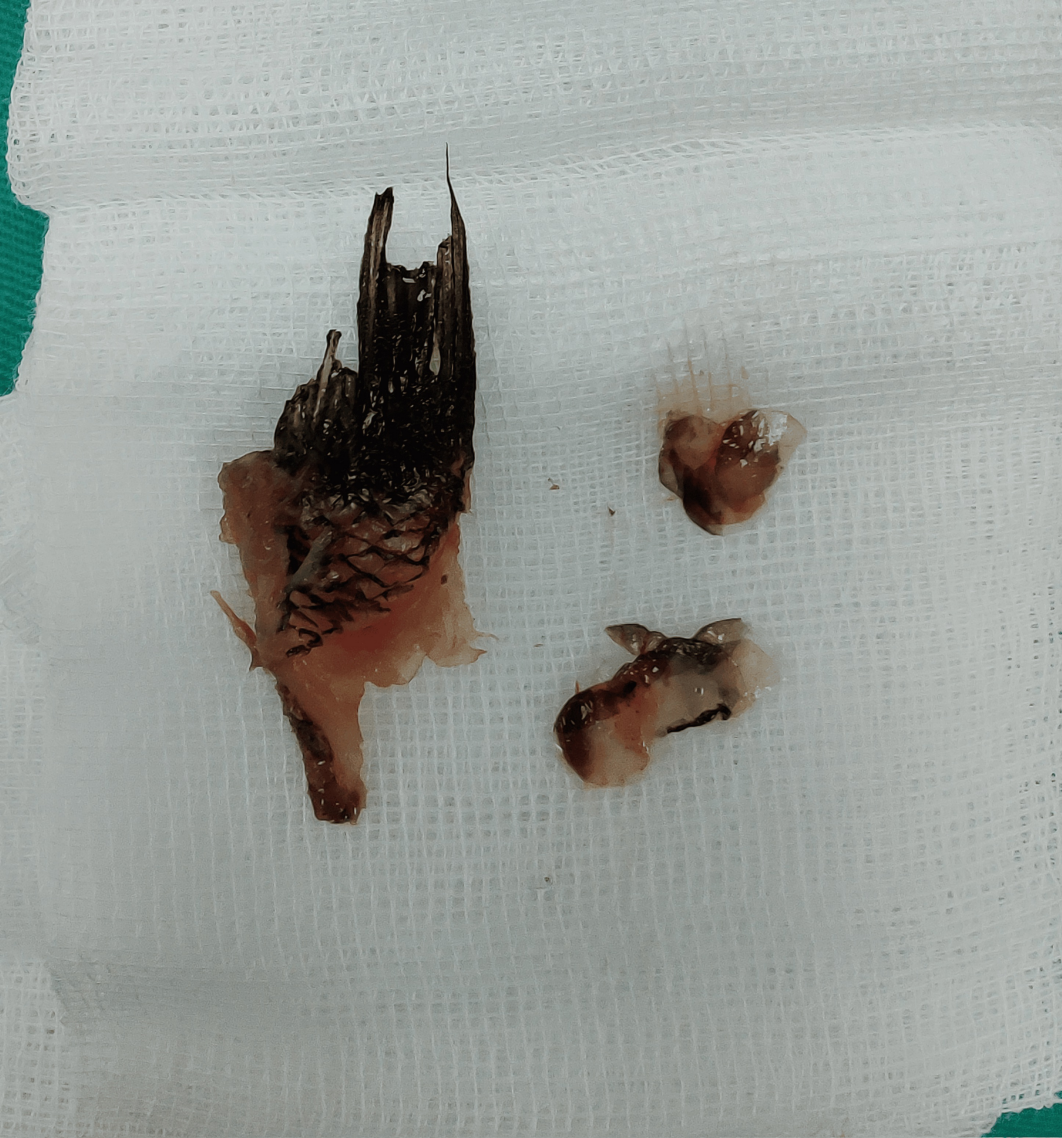
This illustrates the fragments of the fish's tail that broke off during removal attempts. It has thorn-like, sharp bony spikes that caused extensive laryngopharyngeal mucosal injury.

Several attempts at live fish removal were made using Magill forceps, but its vigorous movement thwarted all efforts of grasping it. Furthermore, fragments of the fish's tail broke off during removal, and this heightened the risk of retained fish parts, complicating the extraction process. The fragments of the broken fish's tail are shown in Figure 1.

**Figure 2:**
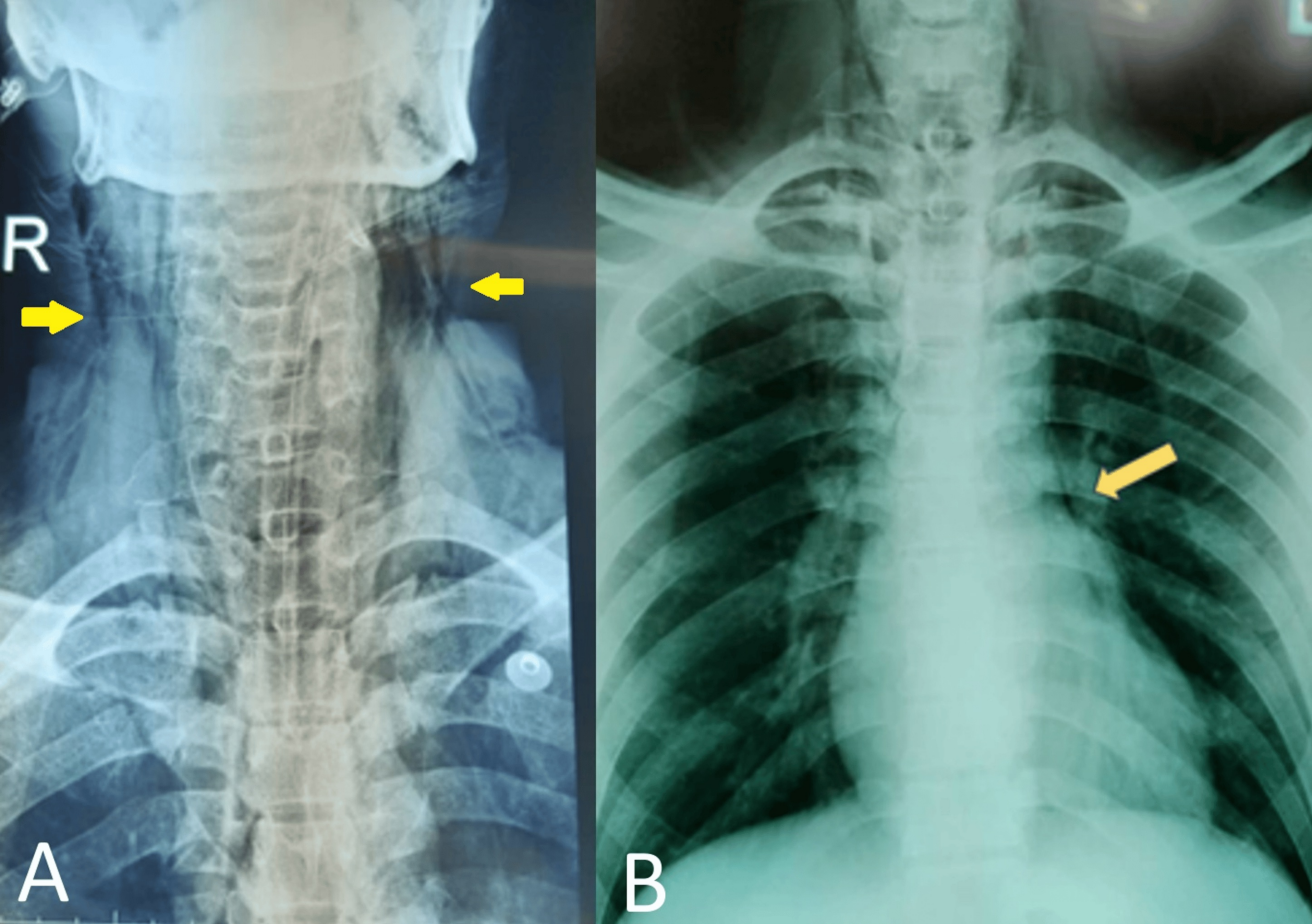
These are the plain radiographs of the patient suggestive of hollow viscus perforation. (A) Extensive subcutaneous emphysema over the neck was noted as marked with a yellow arrow. (B) Pneumomediastinum is highlighted with a yellow arrow.

**Figure 3:**
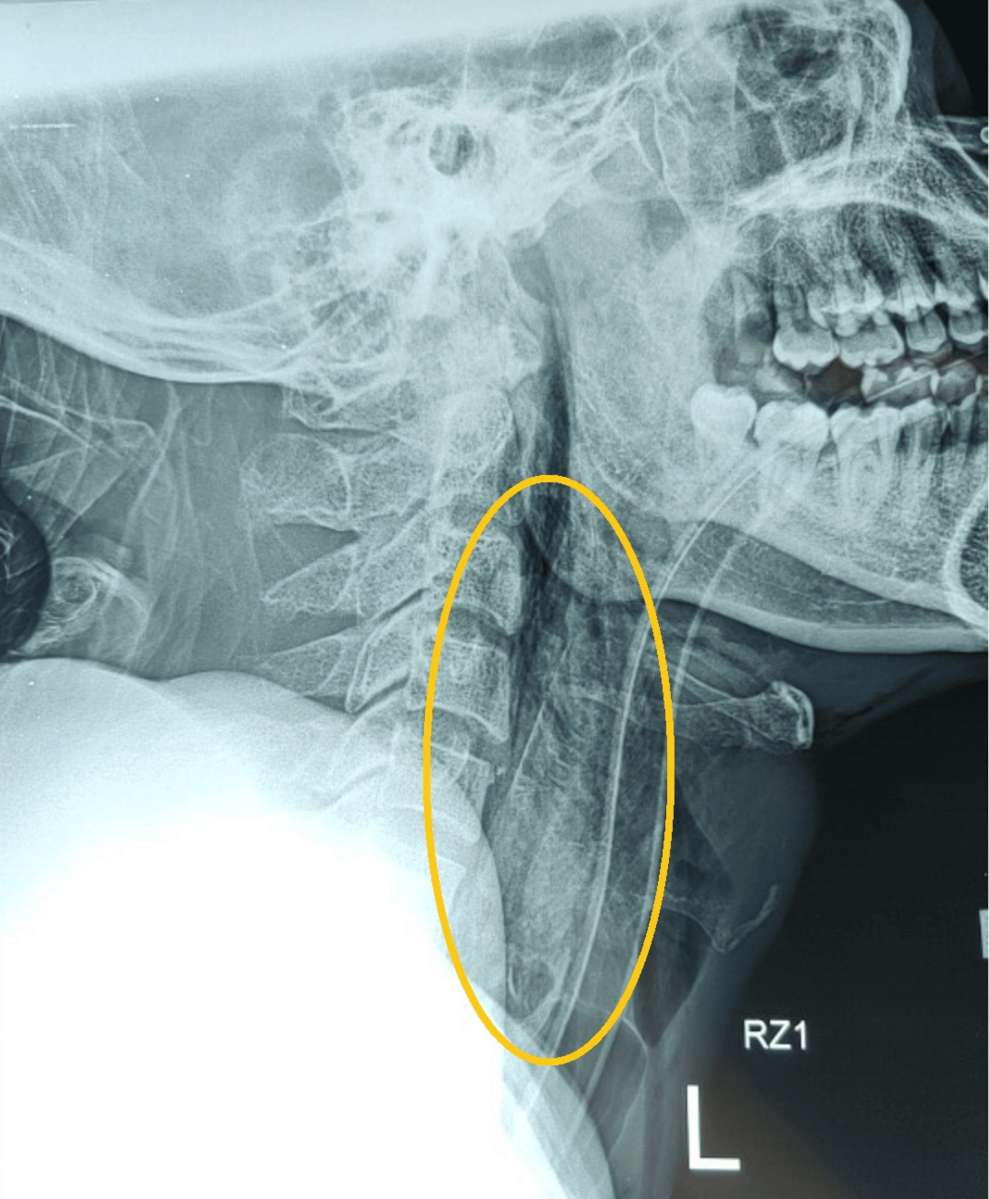
This figure displays a longitudinal opacity bearing resemblance to a fish's vertebrae. Positioned posterior to the airway, its tip extends beyond the cricoid cartilage. The outline of the fish is highlighted in a yellow circle.

At this juncture, the patient developed bilateral neck crepitation. Plain radiography as shown in Figure 2 confirmed the presence of pneumomediastinum and subcutaneous emphysema over the neck. Lateral plain film as shown in Figure 3 identified the location and depth of the fish in the esophagus. This raised the suspicion of hollow viscus perforation, and further attempts of removal were halted. Considering the radiological and interventional limitations of a non-specialist center, the decision was made to transfer the patient to the state hospital for multidisciplinary care. Intravenous dexamethasone was given to reduce laryngeal edema and broad-spectrum antibiotics as prophylactic management to reduce the risk of developing mediastinitis and abscess.

**Figure 4:**
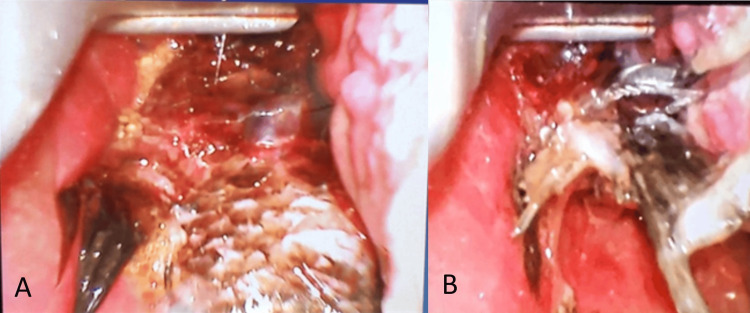
These are images of a video laryngoscope demonstrating the partially visualized fish body and tail in the laryngopharynx which appeared edematous. Image A shows the fish's tail in the hypopharynx. Image B demonstrates the compromised fish tail, after failure of retrieval using Magill forceps.

At the state hospital, a video laryngoscope demonstrated that the fish had transitioned from its previously animated state to limpness likely due to asphyxiation, as shown in Figure 4. Ironically, this had improved the visualization of the pharyngoesophageal junction. Various techniques of removal were employed, including passing through a Foley catheter into the esophagus and intermittently deflating the endotracheal tube cuff to facilitate removal. But all attempts proved futile because the sharp fish fins had penetrated the surrounding laryngopharyngeal mucosa. A quick decision was made to bring the patient to the operation theatre to expedite emergency removal.

Under the controlled environment in the operating theatre, utilizing a direct laryngoscope, the fish's head was seen impacted against the cricoid bone and cricopharyngeus muscle. To mobilize the fish, its sharp fins were dislodged from the surrounding mucosa. Ample lubrication was applied. Once the fish's body was liberated from the surrounding mucosa, it was rotated along its axis. This freed the fish's head from the upper esophagus, and it was successfully removed.

**Figure 5:**
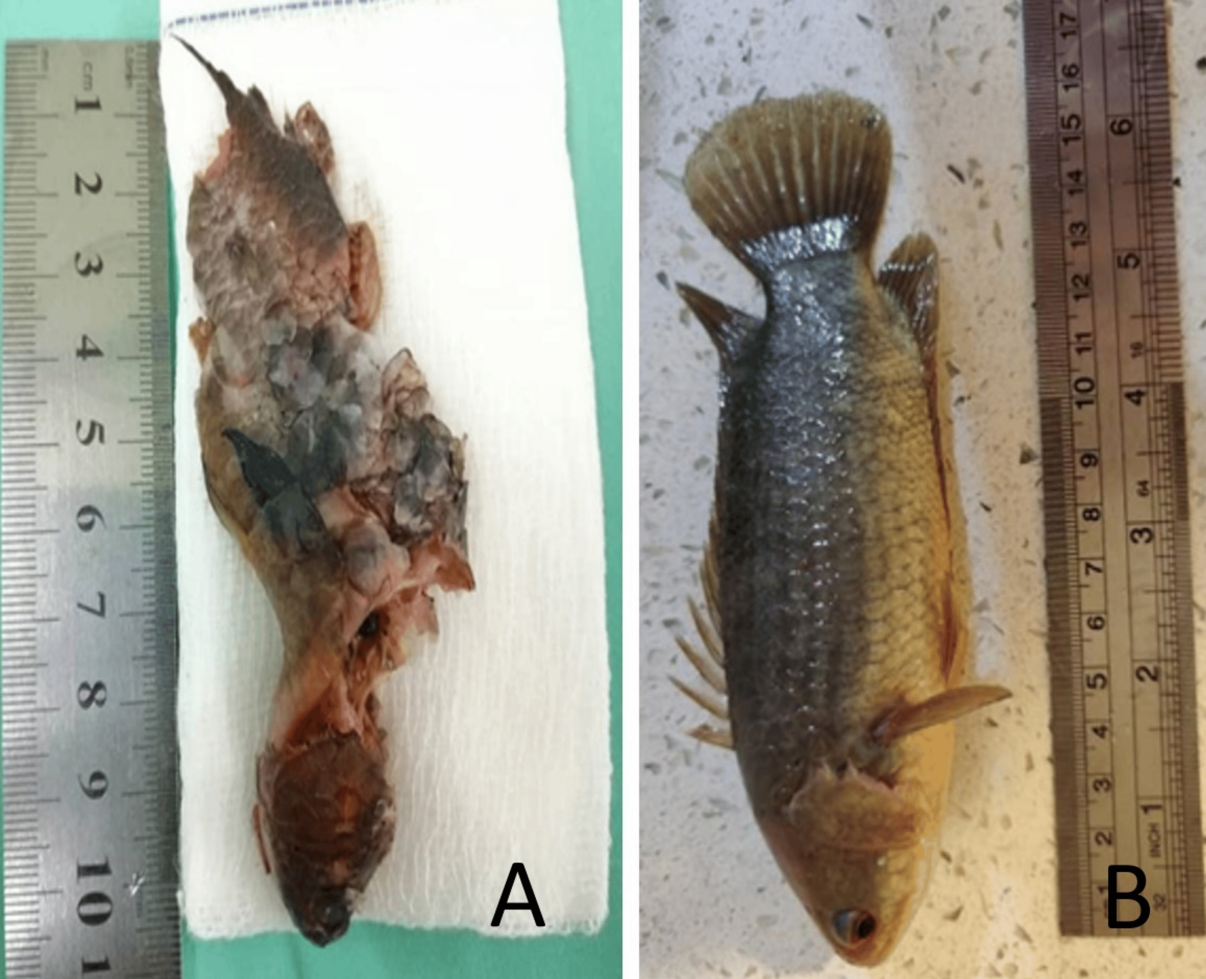
This presents two images. Photo A showcases the fish that was successfully removed. Measuring at least 11 cm in length, this tail-deficient specimen is identified as a climbing perch. Photo B depicts another fish of the same species, demonstrating a comparable size for reference.

The fish was identified as *Anabas testudineus*, commonly known as a climbing perch. It measured 11 cm in length with a compromised tail as shown in Figure 5. After removal, the aerodigestive tract was assessed endoscopically. The laryngopharynx was edematous but remained intact. Esophagoscopy showed a linear laceration over the upper esophageal region, 16-18 cm from the upper incisor. Otherwise, the esophageal mucosa appeared healthy and pink. Areas of punctate hemorrhage were compressed using adrenaline-soaked ribbon gauze to achieve hemostasis.

The patient was extubated on postoperative day 1 (POD 1) and saturated well under room air. The subcutaneous emphysema over the neck resolved, and he tolerated enteral feeding via nasogastric tube. Endoscopic reassessment of the esophageal perforation was postponed until POD 5 due to the patient's close contact with a COVID-19-positive individual, necessitating isolation and observation. Esophagogastroscope (OGDS) later revealed a healed esophageal mucosa. There was no sign suggestive of abscess formation over the neck, mediastinitis, or airway injury. The patient made a full recovery and was discharged well within a week of hospitalization.

## Discussion

Ingested FBs represent a common complaint encountered by emergency and otolaryngology teams [1,2]. Among adults, approximately 90% are due to fish bones [1]. While most smaller FBs can pass through the gastrointestinal tract spontaneously, more sizeable FBs such as live fishes can get impacted in the throat [1-7].

Instances of live fish lodged in the aerodigestive tract are rare and occur mostly in developing countries [3-10]. The climbing perch is an amphibious fish and a common culprit [3-10,17,18]. Most cases have identified the fish in the oropharynx, nasopharynx, and nasal meatus [3-10]. However, in this case, the fish was forcefully pulled by the fishing rod and propelled into the patient's mouth at high speed. This distinct scenario caused the fish to become lodged deeper in the laryngopharynx and upper esophagus and not be visualized upon oropharyngeal inspection. As the fish weakened over time, the tail flopped forward to cover the glottis, causing upper airway obstruction. 

The foremost priority is airway protection. When spontaneous expulsion does not occur, patients are at risk of asphyxiation and death [3-10]. In this case, the long blade of Mac 4 expedited the search for the epiglottis, affording clear visualization of the Cormack-Lehane grade 1 glottic view, ensuring a smooth endotracheal intubation. If intubation failed, emergent cricothyroidotomy would be considered. 

This case presented unique obstacles in removal. Initially, the fish fiercely resisted capture. Its fin penetrated the surrounding mucosa and caused bleeding. Its head was lodged against the cricopharyngeus muscle [19]. This area forms the upper esophageal sphincter and is a common site of FB impaction [11,19]. Eventually, removal was successful by grasping the fish's body using the Magill forceps and retrieving it in an axial rotational direction. This approach helped to dislodge the fish's fins and spine from the mucosa, freeing the fish from the esophagus and allowing its removal as a whole. If the endoscopic approach proved futile, surgical removal via lateral neck incision would be made to facilitate cervical esophagotomy and FB removal [16]. The aerodigestive tract must also be thoroughly checked to exclude retained residual parts of the fish, which can migrate through parapharyngeal spaces and cause chronic inflammation [20].

The fish must be removed because it can cause esophageal perforation [11-16]. Esophageal perforation can be difficult to diagnose due to its vague presentation [11-16]. However, the clinical diagnosis in this case was achieved with a high index of suspicion [11-16]. This is considering the presence of a large, vigorously moving FB in the esophagus and the development of subcutaneous emphysema. Hence, combined with the intention to expedite surgical removal, computed tomography was not performed as a clinical diagnosis was established in advance. 

The management of esophageal perforation depends on its location, the severity of sepsis, and the timing of diagnosis [11-15]. Conventionally, operative management including emergency thoracotomy, debridement, and esophageal repair is the mainstay treatment especially in mid- and distal esophageal perforation [11-16]. However, in this case, the impacted fish caused upper esophageal perforation, which generally causes less severe sepsis when compared to mid- and distal perforation [2,11-16]. Furthermore, an endoscopic assessment confirmed small, well-defined tears with no compromise to esophageal tissue quality [11-15]. Barium study was not performed because if leakage persists, it may lead to chemical mediastinitis [11-14]. Oral feeding was also prohibited until healing was evident [14]. With the early introduction of antibiotics, prompt FB removal, and no sign of local complication or sepsis, non-operative management was opted for, and the patient recovered well.

## Conclusions

While ingestion of FB is a common occurrence, having a large live fish in lodge in the pharynx and upper esophageal sphincter is life-threatening and can cause aerodigestive tract injury. Clinicians should anticipate the technically challenging removal process and hence consider the need for early multidisciplinary intervention. Definitive airway must be secured emergently. Clinicians must hold a high index of suspicion for hollow viscus perforation and consider the indication for urgent FB removal and repair of perforation. As both airway obstruction and esophageal perforation carry a high mortality risk, the emergency team must seek interdisciplinary coordination in critical management, ventilation support, and endoscopic and surgical intervention.
